# Identification of novel DNA hypermethylation of the adenylate kinase 5 promoter in colorectal adenocarcinoma

**DOI:** 10.1038/s41598-021-92147-6

**Published:** 2021-06-16

**Authors:** Bokyung Ahn, Yang Seok Chae, Soo Kyung Lee, Moa Kim, Hyeon Soo Kim, Ji Wook Moon, Sun-Hwa Park

**Affiliations:** 1 Department of Pathology, Asan Medical Center, Seoul, Republic of Korea; 2 Department of Pathology, Korea University Anam Hospital, Korea University College of Medicine, Seoul, Republic of Korea; 3 Medicine and Life Sciences, Journal, Springer Nature, Seoul, Republic of Korea; 4 Institute of Human Genetics, Department of Anatomy, Korea University College of Medicine, Seoul, Republic of Korea; 5 BK21Plus Medical Science, Korea University College of Medicine, Seoul, Republic of Korea

**Keywords:** Cancer, Cell biology, Genetics, Biomarkers, Gastroenterology, Molecular medicine

## Abstract

Adenylate kinase 5 (AK5) belongs to the adenylate kinase family that catalyses reversible phosphate transfer between adenine nucleotides, and it is related to various energetic signalling mechanisms. However, the role of AK5 in colorectal cancer (CRC) has not been reported. In this study, AK5 was significantly hypermethylated in CRC compared to adjacent normal tissues (P < 0.0001) and normal tissues (P = 0.0015). Although the difference in mRNA expression was not statistically significant in all of them, the selected 49 cases of CRC tissues with AK5 hypermethylation with the cut off value of 40% showed a significant inverse correlation with mRNA expression (P = 0.0003). DNA methylation of AK5 promoter significantly decreased and AK5 expression recovered by 5-aza-2′-deoxycytidine, DNA methyltransferase inhibitor in CRC cell lines. In addition, AK5 promoter activity significantly decreased due to DNA methyltransferase, and it increased due to 5-aza. Moreover, AK5 regulated the phosphorylated AMPK and mTOR phosphorylation and inhibited the cell migration and cell invasion in CRC cell lines. Furthermore, low AK5 expression is associated with poor differentiation (P = 0.014). These results demonstrate that the AK5 promoter is frequently hypermethylated and induced methylation-mediated gene down-regulation. AK5 expression regulates AMPK/mTOR signalling and may be closely related to metastasis in colorectal adenocarcinoma.

## Introduction

The causes of colorectal cancer (CRC) are heterogeneous and include multistep tumorigenesis caused by genetic and epigenetic variations^1,2^. Genetic variation can occur via APC, K-ras, p53, and beta-catenin, and it regulates abnormalities in cell adhesion, signalling, angiogenesis, and apoptosis^3^. Epigenetics include inheritable changes in gene expression without DNA sequence changes, and they include DNA methylation, histone modification, and non-coding RNAs^4^. Epigenetic changes are heritable and reversible, and they are reportedly involved in the development and progression of various diseases like cancer^5^. In particular, the down-regulation of tumour suppressor genes by DNA hypermethylation and over-expression of oncogenes by DNA hypomethylation are typical examples^6^. In CRC, epigenetic variation, which is clearly distinguished from existing genetic variation, has been reported as an important mechanism for cancer development^4^. Previous studies showed that CDH1, P16, hMLH1, and various tumour suppression genes involved in several signal pathways are inactivated by DNA hypermethylation during colorectal tumorigenesis^7,8^. Various panels of CRC-specific DNA hypermethylation genes were named CpG island methylator phenotype (CIMP)^9^. These CIMP markers were used to constitute factors for the development and prognosis of the serrated pathway in CRC and were also used to investigate this pathway via clinical trials^10^. However, their clinical uses have been very limited.

AK5, an isoenzyme 5 of the adenylate kinase (AK) family, is located on chromosome 1p31.1. It catalyses the reversible transfer of the terminal phosphate group between ATP and AMP to two molecules of ADP, and it maintains cellular energy homeostasis and regulates cell signalling^11^. Adenylate kinase (AK) is known to have three domains consisting of a core domain, AMP binding domain (AMPbd), and a Lid domain (LID)^12^. It can exist in any cell in the organism, and it plays an important role in cell energy metabolisms and nucleotide homeostasis^13^. AKs are involved in the regulation of cell differentiation, maturation, death, and carcinogenesis, and they can act as intermediates in the AMPK signalling pathway^11,14^. Nine human AK protein family genes have been identified, and AK1 and AK2 have been well studied in the context of cancer^15–17^. Recently, one study reported aberrant AK5 gene hypermethylation in breast cancer cells that led to gene silencing^18^. Another study showed a conflicting result that AK5 induces proliferation and autophagy while inhibiting apoptosis in gastric cancer^19^. There is little information on AK5, and no previous study has investigated the role of AK5 in CRC.

This study aims to investigate a novel CRC-specific gene that is transcriptionally silenced by DNA hypermethylation. CRC tissue and normal tissue were analysed using a methylation chip array, revealing the possible target gene “AK5”. The relationship between DNA methylation and AK5 expression was analysed in CRC tissues and CRC cell lines. In addition, we modulated AK5 expression in the CRC cell line to observe how it affects the functional process of CRC cells. Furthermore, AK5 expression in CRC tissue was analysed for correlation with various clinicopathological characteristics.

## Results

### AK5 is significantly hypermethylated in CRC

**Figure 1:**
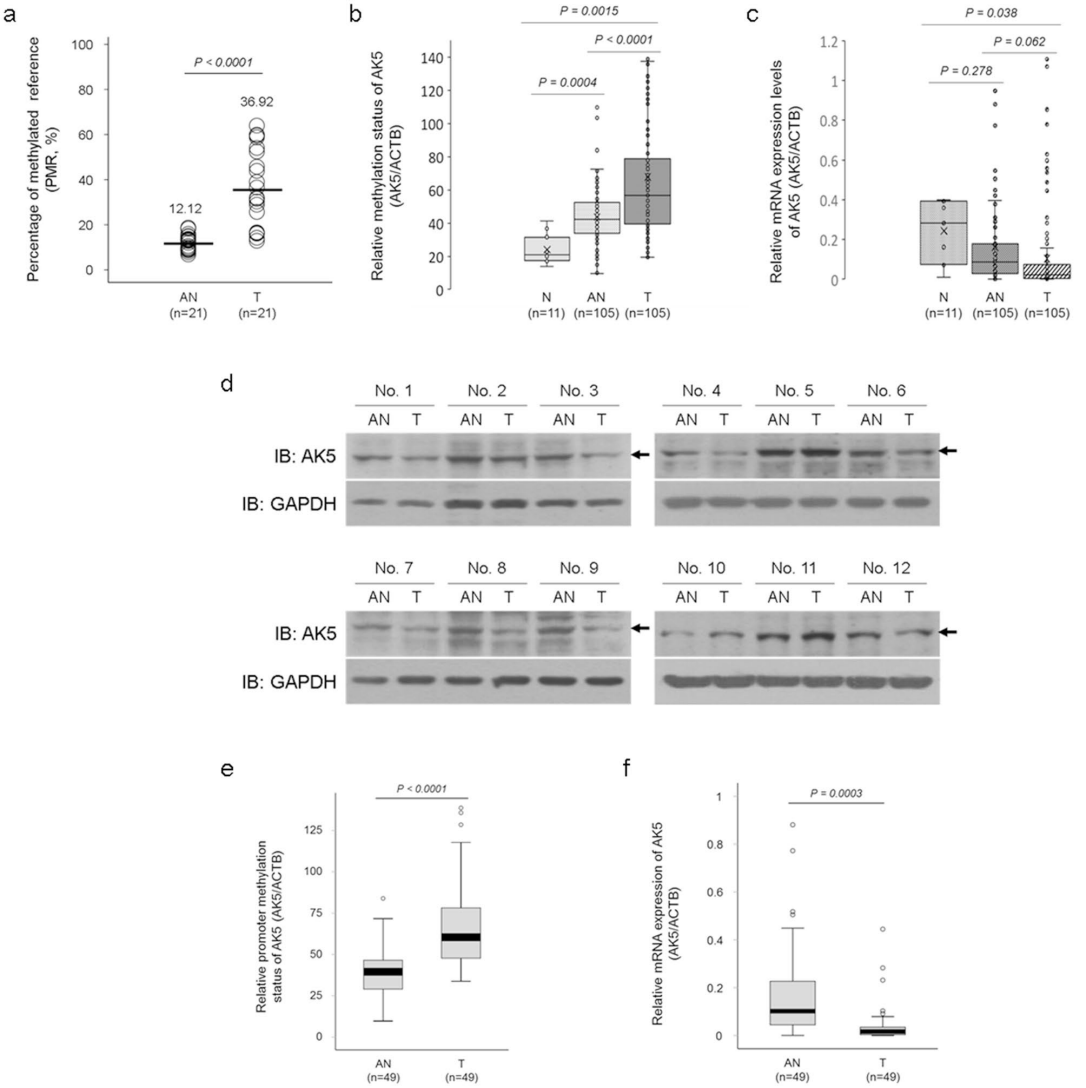
AK5 methylation status and expression levels in CRC tissues and adjacent normal tissues. The AK5 methylation status in 105 paired CRC and adjacent normal tissues and eleven normal tissues was assessed using methylation bead chip array and qMSP. (**a**) The chip array results showed that AK5 is hypermethylated in 21 CRC tissues compared to adjacent normal tissues. (**b**) The AK5 gene is dramatically and significantly hypermethylated in 105 adjacent normal tissues and 105 CRC tissues compared to normal tissues. (**c**) AK5 mRNA expression decreased in the adjacent normal tissues compared to the normal tissues and decreased in the CRC tissues compared to the adjacent normal tissues, but the effect was not statistically significant. (**d**) AK5 protein expression decreased in CRC tissues compared to adjacent normal tissues. (**e**) AK5 hypermethylation was significant in CRC tissues compared to paired adjacent normal tissues in 49 CRC tissues with a cut off value of 40%. (**f**) In 49 CRCs, AK5 mRNA expression significantly decreased in CRC compared to 49 paired adjacent normal tissues. Full-length bolts images or original gel images of AK5 protein expression are presented in Figure S5a. * P-values of < 0.05 were considered statistically significant. *PMR* percentage of methylated reference, *AN* adjacent normal tissue, *T* colorectal cancer tissue, *N* normal colon tissue, *IB* immunoblot.

Aberrant AK5 promoter methylation was discovered using a genome-wide methylation profiling screen using a chip array in 21 CRCs and 21 paired adjacent normal tissues (Fig. 1a)^9^. We performed the qMSP in 105 CRC, 105 adjacent normal, and eleven normal colon tissues to confirm the array results for AK5 DNA methylation. AK5 was significantly hypermethylated in CRC tissues compared to adjacent normal tissue (P < 0.0001), and it was hypermethylated in adjacent normal tissues compared to normal tissues (P = 0.0004, Fig. 1b). We also confirmed AK5 mRNA expression in the same samples using real-time PCR. AK5 mRNA expression in normal tissues gradually decreased in adjacent normal tissues and CRC tissues, but the change was not significant (Fig. 1c). AK5 protein expression decreased in eight out of twelve cases (about 67%) in CRC tissues compared to adjacent normal tissues (Fig. 1d). AK5 methylation status and mRNA expression were reanalysed in 49 CRC tissues with a cut off value of 40% of AK5 hypermethylation compared to paired adjacent normal tissues (P < 0.0001, Fig. 1e). AK5 mRNA expression was significantly decreased in CRC tissues compared to adjacent normal tissues (P = 0.0003, Fig. 1f). The results suggest that AK5 DNA methylation and expression are inversely correlated in CRC tissues.

### AK5 is demethylated and expression is restored by 5-aza in CRC cells

**Figure 2:**
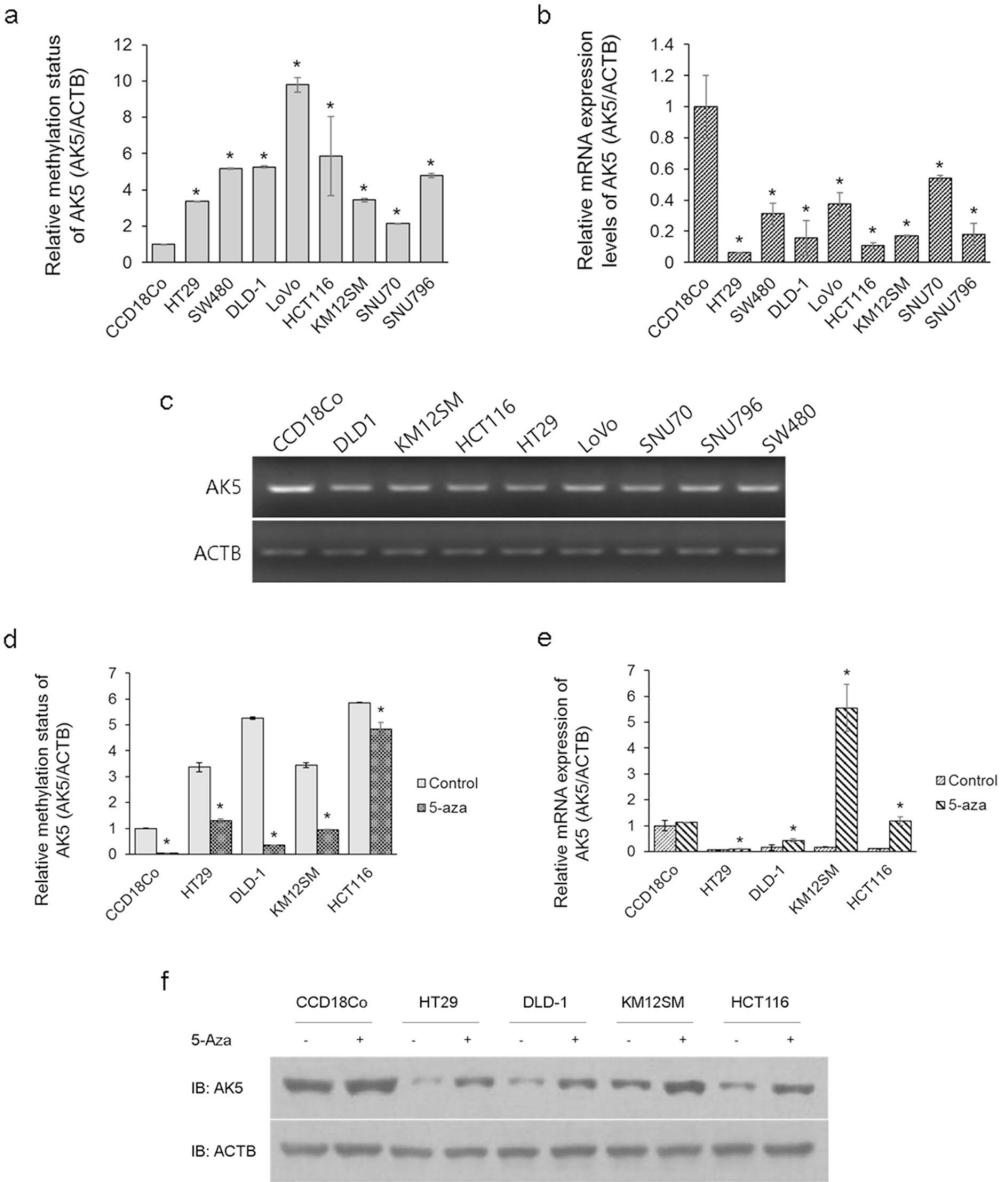
AK5 methylation and expression changes after treatment with 5-aza in CRC cell lines and a normal colon fibroblast cell line. Methylation status, and AK5 expression levels were observed using qMSP, real-time PCR, and immunoblot analysis. (**a**) AK5 was significantly hypermethylated in eight CRC cells compared with normal colon cells. (**b**,**c**) AK5 mRNA expression decreased in eight CRC cells compared to the normal colon cells. (**d**) AK5 DNA methylation decreased in four CRC cells and the normal colon cells after 5-aza treatment. (**e**) AK5 mRNA expression increased in four CRC cells after 5-aza treatment. (**f**) AK5 protein expression increased in four CRC cell lines due to 5-aza. GAPDH or ACTB were used the loading controls. Original gel images of AK5 protein expression are presented in Figure S5b. *P-values of < 0.05 were considered statistically significant. IB, immunoblot.

To identify the relationship between DNA methylation and AK5 expression, we performed qMSP and real-time PCR in one normal colon cell sample (CCD18Co), six colon cancer cell samples (HT-29, SW480, DLD-1, Lovo, HCT116, KM12SM), and two rectal cancer cell samples (SNU-70, SNU-796). The basal DNA methylation of AK5 significantly increased in the eight CRC cells compared to the normal colon cells (Fig. 2a). Furthermore, AK5 mRNA expression decreased in CRC cells (Fig. 2b,c). The effect of methylation on expression was determined after treating with 5-aza for 3 days in normal colon cells (CCD18Co) and four CRC cells (HT-29, DLD-1, KM12SM, and HCT116). The results showed 5-aza’s demethylating effect on AK5 in normal colon cells and four CRC cells (Fig. 2d), and we recovered the mRNA and protein expression in four CRC cells (Fig. 2e,f). These results suggest that AK5 expression is regulated by DNA methylation in CRC cells.

### AK5 promoter hypermethylation decreases promoter activity

**Figure 3:**
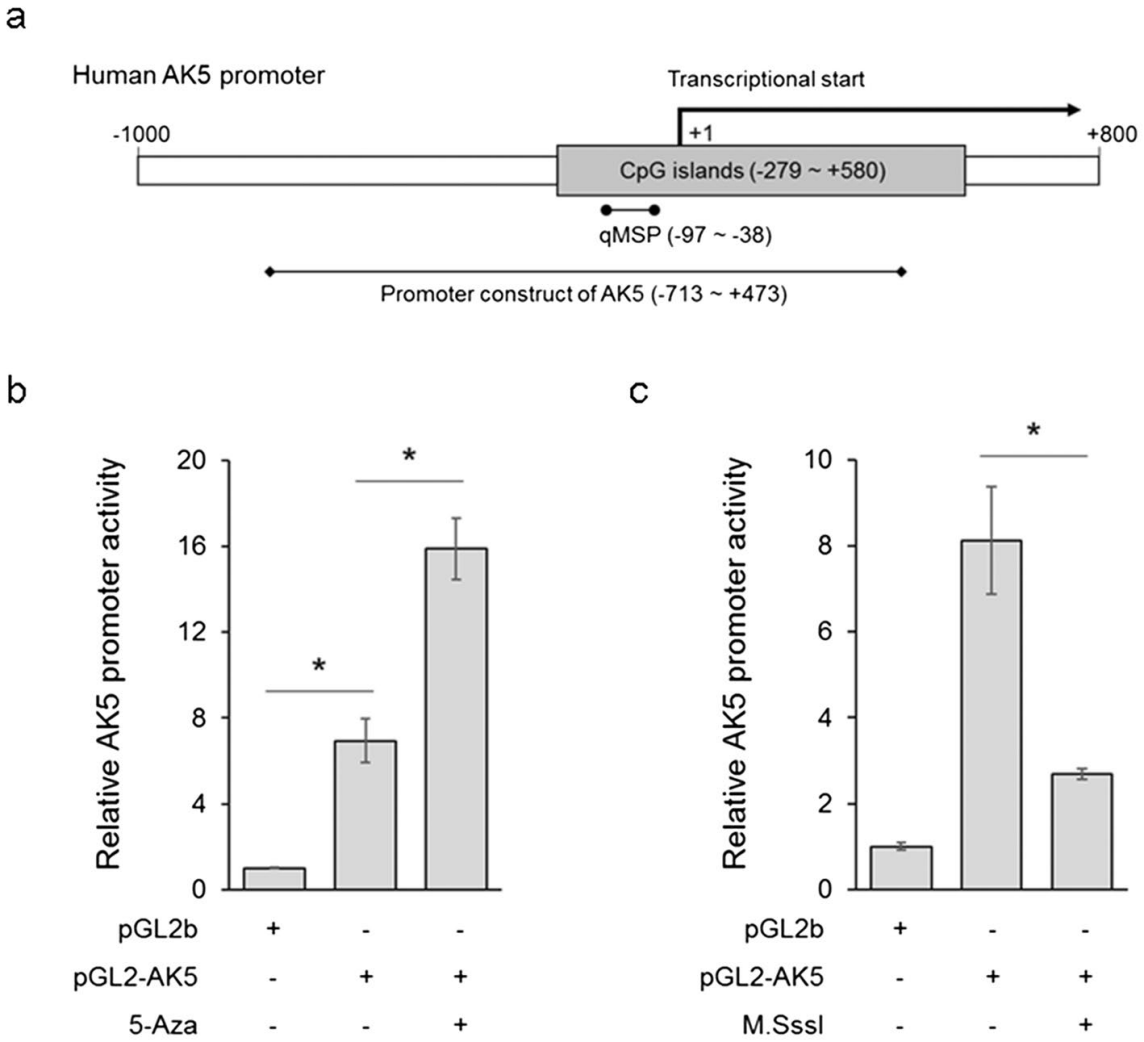
The activity of AK5 promoter regions in CRC cells. The AK5 promoter activity was observed using luciferase assay in DLD-1 cells. (**a**) The human AK5 promoter structures are schematically shown including the designed region of the AK5 promoter construct and AK5 qMSP primer design site containing the CpG island. (**b**) The pGL2-AK5 promoter is highly activated compared to pGL2b. The pGL2-AK5 promoter is significantly activated by 5-aza. (**c**) The AK5 promoter methylated by M.SssI showed significantly decreased promoter activity compared to the normal AK5 promoter in DLD-1 cells. pGL2b luciferase vector was used as the negative control. *P-values of < 0.05 were considered statistically significant.

To examine the activity of the AK5 promoter construct, AK5 promoters were cloned into the pGL2b luciferase vector, and the luciferase activity was measured after transfection with luciferase vectors for 1 day in DLD-1 cells. Figure 3a shows the human AK5 promoter structure and the promoter construct design of the AK5 and qMSP primers. Higher promoter activity was observed when the pGL2-AK5 was transfected compared to when pGL2b was transfected. The AK5 promoter activity was evaluated using 5-aza, a demethylating agent, in DLD-1 cells (Fig. 3b). The AK5 promoter construct form methylated by CpG methyltransferase (M.SssI) presented less activity than the normal promoter construct (Fig. 3c). These results demonstrate that the AK5 promoter is under strict regulation by DNA methylation.

### AK5 inhibits the cell migration and invasion via AMPK/mTOR signal pathway in CRC

**Figure 4:**
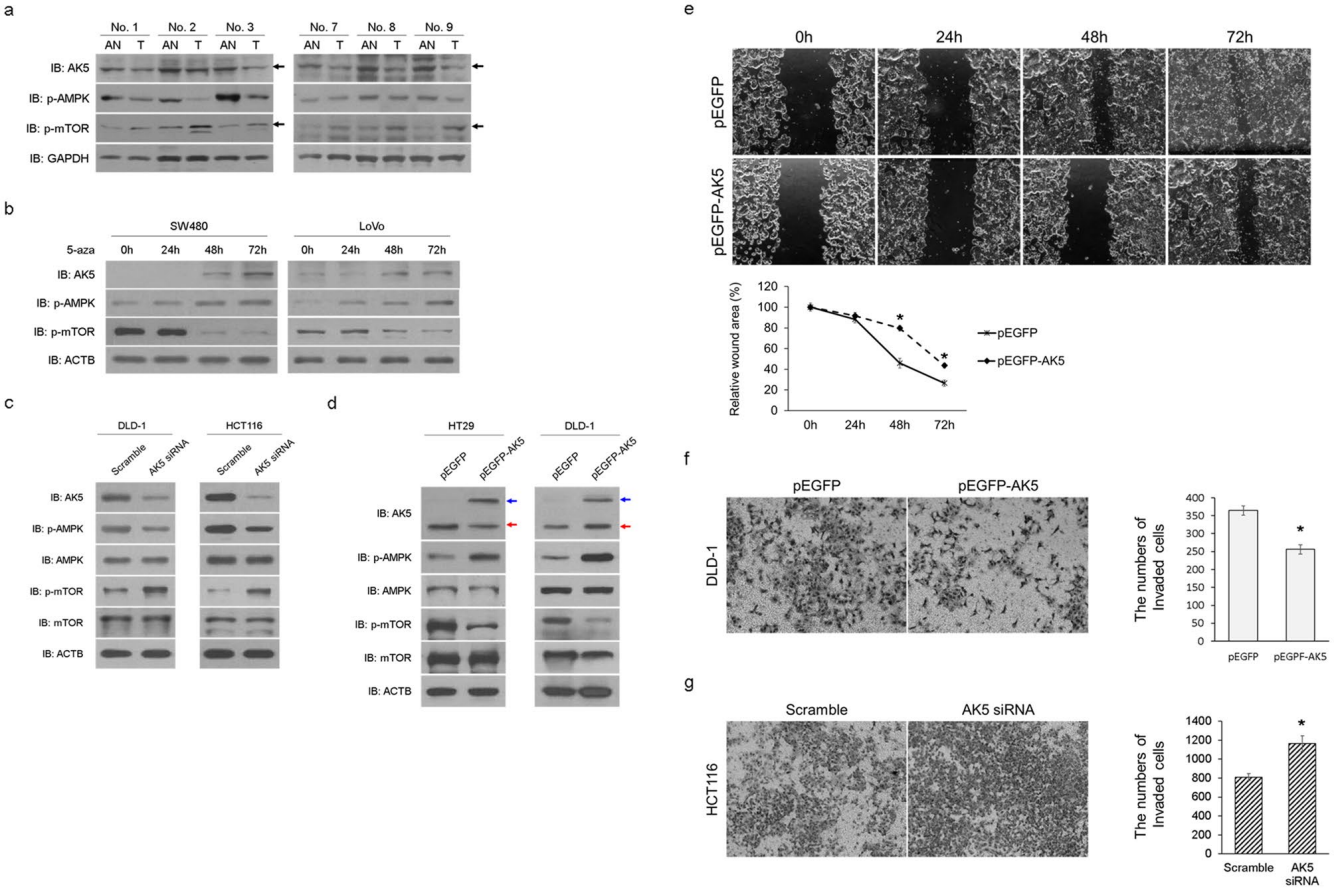
The role of AK5 in the AMPK/mTOR signal pathway in CRC cells. AK5 protein expression, phosphorylated AMPK, and mTOR phosphorylation levels were investigated using immunoblot analysis in three paired CRC tissues and adjacent normal tissues. (**a**) AK5 and AMPK phosphorylation decreased in CRC tissues compared to adjacent normal tissues. However, phosphorylated mTOR increased in CRC. AK5 protein expression, phosphorylated AMPK, and mTOR phosphorylation were investigated using immunoblot analysis in CRC cell lines after treatment with 5-aza for 1–3 days. (**b**) 5-aza increased AK5 expression in a time-dependent manner in SW480 and LoVo cells. AMPK phosphorylation increased for 2 days before decreasing 3 days after 5-aza treatment. Phosphorylated mTOR was decreased by 5-aza in both CRC cells. (**c**) AMPK phosphorylation decreased and mTOR phosphorylation increased when AK5 expression was inhibited by AK5 siRNA transfection into DLD-1 and HCT116 cells for 2 days. (**d**) AK5 overexpression induced AMPK phosphorylation and decreased phosphorylated mTOR in HT-29 and DLD-1 cell lines after transfection with the overexpression vector for 2 days. The blue arrow shows AK5-GFP, and the red arrow shows only AK5. (**e**) The migration of HT-29 cells was significantly reduced after 48 h in AK5 overexpression cells transfected with pEGFP-AK5 compared to pEGFP transfected cells. (**f**) The invasion of DLD-1 cells was significantly reduced after 24 h in AK5 overexpressing cells transfected with pEGFP-AK5 compared to pEGFP transfected cells. (**g**) The invasion of HCT116 cells was significantly increased after 24 h in AK5 knockdown cells transfected with AK5 siRNA compared to scramble siRNA transfected cells. Full-length bolts images or original gel images of AK5 protein expression are presented in Figure S5a, S5c, S5d, S5e and S5f. Original magnification: × 200. GAPDH or ACTB was used as loading controls. *IB* immunoblot, *AN* adjacent normal, *T* colorectal cancer, *5-aza* 5-aza-2′-deoxycytidine. *P-values of < 0.05 were considered statistically significant.

To identify AK5’s regulatory mechanisms for AMPK and mTOR, we performed western blot analysis using target antibodies in CRC tissues and CRC cell lines after treating with 5-aza for 1–3 days. As a result, AK5 protein expression decreased in CRC tissues compared to adjacent normal tissues (Fig. 1d). In addition, phosphorylated AMPK decreased, but it increased mTOR phosphorylation in CRC tissue (Fig. 4a). Moreover, both SW480 and LoVo cells showed AK5 expression recovery after 2 days and increased phosphorylated AMPK and decreased phosphorylated mTOR expression when treated with 5-aza (Fig. 4b). When AK5 expression was inhibited by AK5 siRNA transfection into DLD-1 and HCT116 cells, AMPK phosphorylation decreased and mTOR phosphorylation increased in both cell lines (Fig. 4c). Conversely, when AK5 was overexpressed after transfection with pEGFP-AK5 or pEGFP vector into HT-29 and DLD-1 cells, it was observed that AMPK phosphorylation increased in both cells with AK5 overexpression, while mTOR phosphorylation decreased (Fig. 4d). In addition, it was observed that the pEGFP vector was expressed throughout the cell, and the pEGFP-AK5 vector was expressed in the cytosol (Figure S1).

To examine whether AK5 affects cell migration, and invasion in CRC cells, wound healing and invasion assays were performed in HT-29, DLD-1, and HCT116 cells after transfected with AK5 siRNA or pEGFP-AK5. When AK5 was overexpressed in HT-29, the cell migration was significantly reduced after 48 h (Fig. 4e). Conversely, when the expression of AK5 was down-regulated in DLD-1, it was observed that the cell migration was significantly increased (Figure S2c). The invasion of AK5-overexpressed DLD-1 cells was significantly reduced after 24 h, whereas the invasion of HCT116 cells with down-expressed AK5 was significantly increased (Fig. 4f,g). These results suggest that AK5 inhibits the cell migration and invasion through the AMPK/mTOR signalling pathway in CRC cells.

### AK5 down-regulation is associated with cell differentiation in CRC tissues

**Figure 5:**
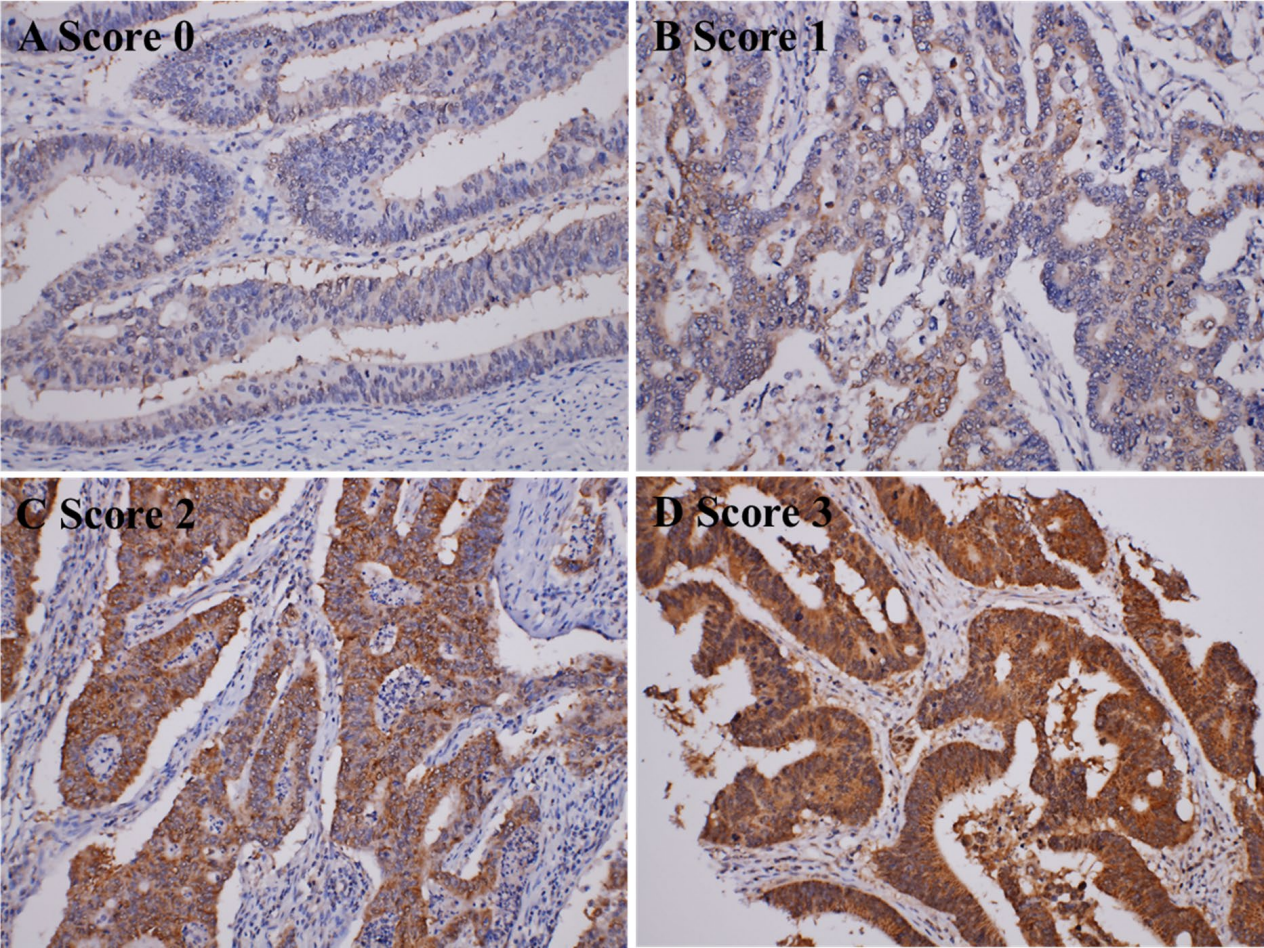
Immunohistochemical representation of the AK5 expression pattern in a CRC tissue microarray. Representative photomicrograph showing different score levels of cytoplasmic AK5 expression in colorectal cancer tissue samples. Original magnification: × 100.

There were 615 cases of CRC patients who underwent surgery for diagnosis and treatment from 2007 January to 2010 December. All slides were histologically evaluated, and a representative tumour area was embedded for tissue microarray and AK5 immunohistochemical staining. AK5 immunostaining was scored using an average cytoplasmic staining intensity of 0 (no expression), 1 (mild intensity), 2 (moderate intensity), and 3 (strong intensity). Scores 0 and 1 were further classified as low expression, and scores 2 and 3 were classified as high expression (Fig. 5). Two hundred and seventy-three cases (44.4%) showed low expression based on AK5 immunostaining, and 342 cases (55.6%) showed high expression. Clinicopathologic factors were evaluated for correlation with AK5 expression, and tumour differentiation showed significant correlation with AK5 expression (P = 0.026, Table 1).

**Table 1:** Association between the AK5 expression and clinicopathologic factors.

Characteristic	No. (n = 615)	AK5 expression		
		Low (%)	High (%)	P-value
		(n = 273)	(n = 342)	
**Age (yr)**				
≤ 60	215	101	114	0.344
> 60	400	172	228	
**Gender**				
Male	359	162	197	0.664
Female	256	111	145	
**Location**				
Colon	369	159	210	0.426
Rectum	246	114	132	
**Differentiation**				
Well	249	94	155	0.026^†^
Moderate	358	172	186	
Poor	8	7	1	
**Size**				
≤ 6 cm	484	211	273	0.446
> 6 cm	131	62	69	
**pT stage**				
pTis, pT1, pT2	145	63	82	0.794
pT3, pT4	470	210	260	
**LVI**				
Absent	522	226	296	0.195
Present	93	47	46	
**PNI**				
Absent	575	261	314	0.058
Present	40	12	28	
**LN metastasis**				
Absent	375	173	202	0.277
Present	240	100	140	
**AJCC stage**				
Stage 1, 2	353	161	192	0.480
Stage 3, 4	262	112	150	
**Distant metastasis**				
Absent	540	239	301	0.861
Present	75	34	41	
**RM**				
Negative	608	270	338	0.935
Positive	7	3	4	
**Local recur**				
Absent	590	263	327	0.652
Present	25	10	15	

*AJCC* American Joint Committee on Cancer, *LVI* lymphovascular invasion, *PNI* perineural invasion, *LN* lymph node, *RM* resection margin. ^†^P*-*values < 0.05 were considered statistically significant.

Survival analyses of AK5 expression along with patient age, gender, tumour size, location, differentiation, stage, lymph node metastasis, resection margin status, and recurrence were performed. Univariate analysis did not show a significant correlation between AK5 expression and survival, but poor differentiation (P = 0.015), larger tumour size (P = 0.003), higher T stage (P = 0.004), LVI (P < 0.001), PNI (P < 0.001), LN metastasis (P < 0.001), positive resection margin (P < 0.001), and DM (P < 0.001) were correlated with poor prognosis. Furthermore, multivariate analysis showed that poor differentiation (P = 0.042), larger tumour size (P = 0.006), LN metastasis (P = 0.014), DM (P < 0.001), and positive resection margin (P = 0.024) were independent poor prognostic factors (Table 2).

**Table 2:** Survival analysis of clinicopathologic factors and AK5.

Clinicopathologic factors	Univariate analysis			Multivariate analysis		
	HR	95% CI	P-value	HR	95% CI	P-value
AK5, high vs. low	0.876	0.551–1.391	0.575			
Age (yr), > 60 vs. ≤ 60	1.179	0.722–1.925	0.511			
Gender, female vs. male	1.381	0.870–2.193	0.171			
Location, rectum vs. colon	0.764	0.470–1.241	0.276			
Differentiation, poor vs. well, moderate	4.208	1.321–13.411	0.015^†^	3.508	1.046–11.763	0.042^†^
Size, > 6 cm vs. ≤ 6 cm	2.126	1.302–3.473	0.003^††^	2.180	1.247–3.809	0.006^†^
pT stage, pT3-4 vs. pT0-2	3.130	1.435–6.828	0.004^†^	1.266	0.543–2.950	0.585
LVI, presence vs. absence	2.653	1.596–4.412	< 0.001^†^	1.483	0.850–2.585	0.165
PNI, presence vs. absence	4.304	2.398–7.726	< 0.001^†^	1.439	0.715–2.894	0.308
LN metastasis, present vs. absent	3.475	2.128–5.673	< 0.001^†^	3.816	1.305–11.160	0.014^†^
AJCC stage, stage 3,4 vs. stage 1,2	4.374	2.588–7.390	< 0.001^†^	0.455	0.132–1.572	0.213
Distant metastasis, presence vs. absence	10.057	6.227–16.241	< 0.001^†^	7.749	4.121–14.571	< 0.001^†^
RM, positive vs. negative	14.665	5.266–40.842	< 0.001^†^	4.102	1.201–14.016	0.024^†^
Local recur, present vs. absent	4.015	1.991–8.095	< 0.001^†^	2.721	1.296–5.711	0.008^†^

*AJCC* American Joint Committee on Cancer; *LVI* lymphovascular invasion, *PNI* perineural invasion, *LN* lymph node, *RM* resection margin, *HR* hazard ratio, *CI* confidence intervals. ^†^P*-*values < 0.05 were considered statistically significant.

## Discussion

In our previous study, we selected the DNA hypermethylation candidate gene “AK5” using a chip array in CRC. AK5 hypermethylation is not involved in 21 top-ranked hypermethylation genes in CRC^9^, but AK5 is significantly hypermethylated in CRC and is involved in the top 30 genes (Table S2). Here, we confirmed the DNA hypermethylation of AK5 in CRC. The DNA methylation status and mRNA expression levels of AK5 did not statistically correlate in 105 CRC cases. However, AK5 hypermethylation with a cut off value of 40% in selective CRC tissues was significantly inversely correlated with AK5 mRNA expression in the same specimens. In these cases, AK5 mRNA expression was significantly correlated with TNM stage (P = 0.044). Unfortunately, AK5 methylation and expression were not significantly correlated with other clinicopathological features (Table S3).

Basal DNA methylation status of AK5 was significantly increased in eight CRC cell samples compared to normal colon cell samples. AK5 mRNA expression also showed a significant decrease in expression in eight CRC samples. However, no significant changes in AK5 methylation or expression were observed with tumour grades or location of CRC cells. In addition, AK5 DNA hypermethylation decreased in four colon cancer cell samples after treatment with 5-aza, a DNA methyltransferase inhibitor, and expression recovery was observed (Fig. 2). Moreover, it was confirmed that the AK5 promoter was significantly activated by 5-aza, and it inactivated DNA methylation (Fig. 3). This result shows that the expression of AK5 in CRC cells is regulated by DNA methylation.

AKs reversibly promote AMP phosphorylation using ATP and AMP as substrates in various signal transductions including AMPK phosphorylation^20^. AMPK is known to act as a cell energy sensor in relation to glucose metabolism^21^. Recently, AMPK has been found to suppress proliferation or metastasis of CRC cells, inducing autophagic cell death by AMPK/mTOR signalling and suppression of CRC cell invasion via the AMPK/Akt/mTOR signal pathway^22–24^. Here, we also observed decreased AK5 expression, AMPK phosphorylation, and increased mTOR phosphorylation in CRC tissues. In CRC cells after treatment with 5-aza, AK5 protein expression recovery, phosphorylated AMPK induction, and mTOR phosphorylation reduction were observed. In addition, AK5 regulated phosphorylated AMPK and mTOR phosphorylation and inhibited the migration and invasion in CRC cells (Fig. 4). However, changes of AK5 expression did not affect the viability in CRC cells (Figure S2a, S2b). These results confirmed that AK5 can affect the metastasis of CRC cells by regulating the AMPK/mTOR signalling mechanism by increasing AMPK activity like other AKs.

Finally, IHC analysis of AK5 was performed using 615 CRC tissues, 25 adjacent normal tissues, and 26 liver and lung metastasized cancer tissues to find the relationship between AK5 expression and clinicopathological features. As a result, it was observed that AK5 expression was significantly correlated with CRC differentiation, but it did not show a significant correlation with other clinicopathological characteristics (Table 1). In addition, it was confirmed that the 615 CRC tissues were associated with poor prognosis, such as degree of differentiation, cancer size, TNM stage, and DM. In addition, multivariate analysis showed that cancer differentiation and size metastasis are independent prognostic factors (Table 2). Furthermore, AK5 expression was compared between adjacent normal and primary CRC and metastatic cancer tissues. Although it was not clinically significant, it was observed that AK5 expression decreased in metastatic cancer tissues compared to primary CRC tissues and adjacent normal tissues (P = 0.068, P = 0.218, respectively, Figures S3, S4). It is expected that significant data may be generated when the number of metastatic cancer tissues increase.

In conclusion, AK5 hypermethylation occurs frequently, and AK5 expression was reduced in CRC. When the cut off value of AK5 hypermethylation was set to 40% or more, it was shown that there was a statistically significant inverse correlation with mRNA expression. In addition, it was confirmed in the CRC cells that AK5 expression was regulated by DNA methylation, and AK5 is involved in cell invasion and migration through AMPK/mTOR signalling by activating AMPK. Moreover, AK5 expression was statistically significantly related to CRC differentiation. Further studies will have to be conducted using more tissue samples from CRC patients to analyse whether AK5 could be used as a prognostic factor for metastasis.

## Materials and methods

### Tissues

One hundred and five CRC and adjacent normal tissue samples and eleven normal colon tissue samples were obtained from the Department of Colorectal Surgery, Korea University Medical Center. Normal tissue samples were collected through laparoscopic surgery with consent from the hernia patient. The tissues for the epigenetic study were used after obtaining approval from the Institutional Review Board of Korea University College of Medicine (IRB No. KU-IRB-13-84-A-1) and informed consent was obtained from all subjects or, if subjects are under 18, from a parent and/or legal guardian. Every fresh tissue sample was frozen in liquid nitrogen after resection and stored in a refrigerator at − 80 °C. Additionally, 676 surgically resected CRC tissues were retrieved from the Department of Pathology, Korea University Anam hospital, from January 2007 to December 2010. Patients who received preoperative treatment and cases with no available block were excluded, and a total of 615 CRC cases, 26 cases of metastases to the liver and lungs, and 25 cases of adjacent normal colon in patients with metastasis were reviewed. All haematoxylin and eosin (H&E) slides of the cases were reviewed by two pathologists (BKA and YSC), and the most representative area was retrieved for tissue microarray. Clinicopathologic data, including age, sex, survival months, survival, tumour location, tumour size, grade, pathologic T (pT) category, pathologic N (pN) stage, lymphovascular invasion (LVI), perineural invasion (PNI), lymph node (LN) status, distant metastasis, and resection marginal status were reviewed. The TNM stages were adjusted to the 8th American Joint Committee on Cancer Staging Manual. All experimental protocols were approved by the Institutional Review Boards of Korea University Anam Hospital (K2018-2161-001). All methods were carried out in accordance with relevant guidelines and regulations. The characteristics of each subject are summarized in Table 3.

**Table 3:** Clinicopathologic characteristics of colorectal cancer patients.

Characteristic	Methylation array subjects	Validation subjects		IHC subjects
	Patients (n = 21)	Non-patients (n = 11)	Patients (n = 105)	Patients (n = 615)
**Age (yr)**				
≤ 60	5	7	28	215
> 60	16	4	77	400
**Gender**				
Male	14	4	66	359
Female	7	7	39	256
**Location**				
Colon	16	11	72	369
Rectum	5		33	246
**Differentiation**				
Well	7		29	249
Moderate	14		73	358
Poor	0		3	8
**Size**				
≤ 6 cm	9		79	484
> 6 cm	12		26	131
**AJCC stage**				
Stage 1, 2	11		46	353
Stage 3, 4	10		59	262
**Distant metastasis**				
Absent	19		88	540
Present	2		17	75
**RM**				
Negative				608
Positive				7
**Local recur**				
Absent				590
Present				25

*AJCC stage* American Joint Committee on Cancer stage, *RM* resection margin.

### Cell lines

One normal colon fibroblast cell line (CCD18Co) and six colon cancer cell lines (HT-29, early stage; SW480, Dukes' type B; DLD-1, Dukes' type C; LoVo, Dukes' type C and stage IV; HCT116 and KM12SM, highly metastasis) were obtained from the American Type Culture Collection (Manassas, VA, USA). Two rectal cancer cell lines (SNU-70 and SNU796) were purchased from Korea Cell Line Bank (Seoul, Korea). CCD18Co cells were cultured in Eagle’s minimum essential medium, and eight CRC cells were cultured in RPMI 1640 and DMEM at 37 °C and 5% CO_2_ atmosphere supplemented with 10% foetal bovine serum (Hyclone, Logan, UT, USA) and 1% penicillin/streptomycin (Hyclone).

### Genomic DNA extraction and sodium bisulfite DNA modification

Genomic DNA was extracted using the QIAamp DNA Mini Kit (Qiagen, Valencia, CA, USA) according to the manufacturer’s recommendations^9^. Genomic DNA was eluted using 100 μL of water and quantified using a NanoDrop ND-100 device (Thermo Fisher Scientific, MA, USA). Genomic DNA (2 μg) was placed in 20 μL of RNase-free water and then modified using the EpiTect Fast DNA Bisulfite Kit (Qiagen). Bisulfite conversion was performed according to the manufacturer’s recommendations. The bisulfite-converted genomic DNA was eluted from the column using 100 μL dH_2_O and stored at − 80 °C until use.

### Methylation bead chip array

Human Methylation 27 Bead Chip (Illumina Inc., CA, USA) is one of the methylation profiling technologies based on bisulfite modification of DNA^9^. This array can provide methylation information at single-base resolution for 27,578 CpG sites spanning more than 14,495 genes. One microgram of bisulfite converted genomic DNA was applied to the bead chip using Illumina-supplied reagents and conditions. After extension, the array was fluorescently stained and scanned, after which the intensities of the M (methylated) and U (unmethylated) bead types were measured. Each methylation data point was represented using fluorescent signals from the M and U alleles. The ratio of fluorescent signals was computed from the two alleles: β value = (max (M, 0))/(|U| + |M| + 100). The β value reflects the methylation level of each CpG site. A β value of 0 to 1.0 indicates the percent methylation from 0 to 100%, respectively.

### Quantitative methylation-specific PCR (QMSP)

The quantitative methylation status in AK5 was confirmed using qMSP with the Applied Biosystems 7500 real-time PCR system (Thermo Fisher Scientific) according to the manufacturer’s recommendations^9^. AK5 methylation primers were designed using the MethPrimer software (http://www.urogene.org/methprimer/) and are shown in Table S1. The PCR reaction was performed using an optical 96-well tray in a final volume of 20 μL. The reaction mixture consisted of 10 μL of 2 × Maxima SYBR Green/ROX qPCR Master Mix (Thermo Fisher Scientific), 250 nM of each primer, and 30 ng of bisulfite-converted DNA template. The QMSP program started at 95 °C for 2 min, followed by 45 cycles at 95 °C for 15 s and 60 °C for 1 min. Each DNA sample was analysed in triplicate. Relative quantification of the amplified gene levels in the bisulfite-converted genomic DNA sample was performed by measuring the threshold cycle (CT) values of AK5 and ACTB. The mean quantity of AK5 was divided by the mean quantity of ACTB for normalization. A known concentration of bisulfite-converted genomic DNA was measured at 1, 1/4, 1/16, and 1/64 via serial dilutions to use as a standard curve for quantification. CpG methyltransferase M.SssI (NEB, MA, USA) modified genomic DNA was used as a positive control according to the manufacturer’s recommendations. DNA methylation according to M.SssI was verified using the restriction enzyme BstUI (NEB).

### mRNA extraction and reverse transcription polymerase chain reaction (RT-PCR)

mRNA was isolated from cells using TRIZOL (Invitrogen, CA, USA)^25^. One microgram of mRNA from each sample was reverse transcribed to cDNA using Moloney murine leukaemia virus RT and random hexamers (Promega, Madison, WI, USA). cDNA synthesis was performed according to the manufacturer’s recommendations. Primers were designed using Primer3 version 0.4.0 (http://primer3.wi.mit.edu/) and are shown in Table S1. mRNA expression was investigated using real-time PCR with an Applied Biosystems 7500 real-time PCR system (Thermo Fisher Scientific) in accordance with the manufacturer’s instructions. Each sample was analysed in triplicate, and the mean quantity of AK5 was divided by the mean quantity of ACTB or GAPDH.

### Chemical treatment

In a previous study, we determined the optimal concentrations of 30 μM or 100 μM 5-aza-2′-deoxycytidine (5-aza, Sigma-Aldrich, Darmstadt, Germany) in CRC cells using an MTT assay^9^. The cells were seeded in 6-well culture plates (SPL life science, Korea) at a density of 1 × 10^5^ cells per well. After 24 h, each cell was treated with 30 μM 5-aza for 48 h at 37 °C and in a 5% CO_2_ atmosphere. After 48 h, each cell was washed three times using PBS (Sigma-Aldrich) before harvesting. The experiments were repeated at least three times.

### Transfection of AK5 siRNA and overexpression vector

A commercial AK5 siRNA (cat. 4392420) and non-target siRNA were purchased from Qiagen, and the pEGFP-AK5 and pEGFP-N1 vectors were purchased from Cosmo Genetech. The construct was verified using electrophoresis on a 0.8% agarose gel and confirmed using DNA sequencing (Cosmo Genetech). Transient transfections of siRNA and expression vector were performed using Lipofectamine 2000 (Thermo Fisher Scientific) according to the manufacturer’s protocol. The cells were cultivated for 24 or 48 h, washed three times in PBS, and harvested. The transfection efficiencies of AK5 and control vector were observed using a fluorescence microscope CKX53 (Olympus, Tokyo, Japan) and ImageView software program. The pEGFP-N1 vector was used as a control. Each extract was assayed three times.

### Promoter luciferase assay

The AK5 promoter primers are shown in Table S1. The amplified PCR product was analysed using agarose gel electrophoresis and cloned into the luciferase promoter pGL2-basic vector (pGL2b, Promega) between the KpnI and HindIII sites. The nucleotide sequence of the pGL2- AK5 promoter construct was verified using DNA sequencing (Cosmo Genetech). The methylated AK5 promoter was artificially modified using M.SssI. The methylated AK5 promoter was digested using KpnI and HindIII and recloned into the pGL2b vector. Cells were grown to 70% confluence in a culture plate, and transient transfection was performed using Lipofectamine 2000 (Thermo Fisher Scientific). Cell extracts were prepared 24 h post-transfection using the luciferase assay as previously described^26^. The pGL2b vector was used as a control. Each extract was assayed at least three times.

### Cell migration and invasion assay

The cell migration was evaluated using a wound-healing assay. 3 × 10^5^ Cells were seeded into 6-well plates and transfection was performed 1 day later. After incubation for 1 day, cell was scratched with a 200 μL sterile micropipette tip and the wells were washed twice with PBS to remove detached cells. The cells were cultured in RPMI-1640 supplemented with 2% FBS to minimize cell proliferation during the period of assay. The scratched image of the same location was captured after 24, 48, and 72 h using microscope CKX53. The migrated area was measured from the captured images using Image J (Ver. 1/52n, NIH, Bethesda, MD, USA). The cell invasion was evaluated using in Transwell Permeable Supports, Polycarbonate Membrane (8 μm pore size, 6.5 mm diameter, Corning Inc., NY, USA) coated with Corning Matrigel Matrix (Corning Inc.). 5 × 10^4^ Cells were seeded in the upper chamber of the transwell and cultured for 1 day. The cells were transfected and incubated for 1 day, and then changed to serum free media in the upper chamber. The lower chamber of the transwell were filled with complete media containing 10% FBS as an attractant. After 24 h, the invaded cells were fixed and stained by Hematoxylin (Sigma-Aldrich) or Giemsa staining solutions (5%, Sigma-Aldrich) for five minutes after removing non-invasive cells. The numbers of invaded cells were counted in five representative fields of membrane under using microscope CKX53.

### Western blot analysis

Total protein was extracted in RIPA buffer containing protease inhibitors (15 mM PMSF, 1 mM NaF, and 1 mM Na3VO4). Protein (30 μg) from whole cell lysates were mixed with 2 × SDS sample buffer and boiled for 10 min at 95 °C. Next, the lysates were separated using SDS-PAGE and transferred to a nitrocellulose membrane (GE Healthcare Life Science, PA, USA). The membrane was blocked with 5% skim milk and incubated with antibodies against AK5 (Abcam, Cambridge, UK), p-AMPK, and p-mTOR (Santa Cruz Biotechnology, Santa Cruz, CA, USA). After washing in TBS containing 0.05% Tween 20, the blots were incubated with horseradish peroxidase-conjugated anti-mouse or anti-rabbit secondary antibodies (Santa Cruz Biotechnology). The protein bands were visualized using a SuperSignal West Dura Extended Duration Substrate (Thermo Fisher Scientific). The ACTB (Sigma-Aldrich) antibody was used to confirm comparable loading.

### Tissue microarray (TMA) and immunohistochemistry (IHC)

TMA was constructed from formalin fixed paraffin embedded tissue blocks with a tissue microarrayer. Two cores with a diameter of 3.0 mm were extracted from selected tumour blocks and rearranged to recipient blocks. The blocks were sectioned to a thickness of 4 um. Sections were deparaffinized for 5 min three times in xylene and rehydrated for 5 min per session in series of alcohol gradients (100%, 95%, 80%, 70% alcohol). Antigen retrieval was performed for ITLN1. Declere (Cell marque, USA) was heated in a pressure cooker for 15 min to retrieve antigens. Next, the container was cooled for 20 min at room temperature. The slide was incubated in a hydrogen peroxide block (Cell marque) for 10 min to reduce non-specific background staining. The slides were washed three times in TBS (pH 7.6) for 5 min and incubated with a Protein Block (Cell marque) at room temperature for 5 min. An anti-AK5 antibody (1:600, NOVUS, USA) was used. The antibodies were incubated for 50 min at room temperature and washed three times in TBS for 5 min. After applying the primary antibody, it was incubated for 10 min in the amplifier. Subsequently, the secondary antibody reaction was achieved using an HRP polymer (GBI detection kit, USA) for 30 min at room temperature. After washing with TBS, the samples were stained using a DAB chromogenic reaction and counter-stained using Mayer’s Hematoxylin (scytec, USA). IHC results were scored by two pathologists and quantified using Leica’s Aperio ImagScope program (V12.4.0.5043).

### Statistical analysis

Statistical significance of the array data was determined using a paired *t* test. The significance of the different methylation status of AK5 among CRC tissues, adjacent normal tissues, and normal tissues was defined using one-way analysis of variance (ANOVA) with IBM SPSS Statistics version 21.0 (IBM Inc., Chicago, IL, USA). All statistical tests were two-sided, and values P < 0.05 were considered to indicate statistical significance.

## Supplementary Information

Supplementary Information.
