# Supplementary material for: Identification of novel DNA hypermethylation of the adenylate kinase 5 promoter in colorectal adenocarcinoma
Source: Sci Rep. 2021 Jun 16;11:12626. doi: 10.1038/s41598-021-92147-6 (PMC8209216; doi:10.1038/s41598-021-92147-6)
Supplement: Supplementary file 1 — Supplementary Information. [file 41598_2021_92147_MOESM1_ESM.docx]

**Supplementary Information**

**Identification of novel DNA hypermethylation of the adenylate kinase 5 promoter in colorectal adenocarcinoma**

Bokyung Ahn^1,2†^, Yang Seok Chae^2†^, Soo Kyung Lee^3^, Moa Kim^4^, Hyeon Soo Kim^4^, Ji Wook Moon^4,5*^, and Sun-Hwa Park^4*^

^1^Department of Pathology, Asan Medical Center, Seoul, Republic of Korea, ^2^Department of Pathology, Korea University Anam Hospital, Korea University College of Medicine, Seoul, Republic of Korea, ^3^Medicine & Life Sciences, Journal, Springer Nature, Seoul, Republic of Korea, ^4^Institute of Human Genetics, Department of Anatomy, Korea University College of Medicine, Seoul, Republic of Korea, and ^5^BK21Plus Medical Science, Korea University College of Medicine, Seoul, Republic of Korea

^†^ These authors contributed equally to this study

^*^Correspondig: [mjw6132@korea.ac.kr](mailto:mjw6132@korea.ac.kr) (J.W. Moon); [parksh@korea.ac.kr](mailto:parksh@korea.ac.kr) (S.-H. Park)

**Conflict of interest:** The authors declare to have no competing interests.

**
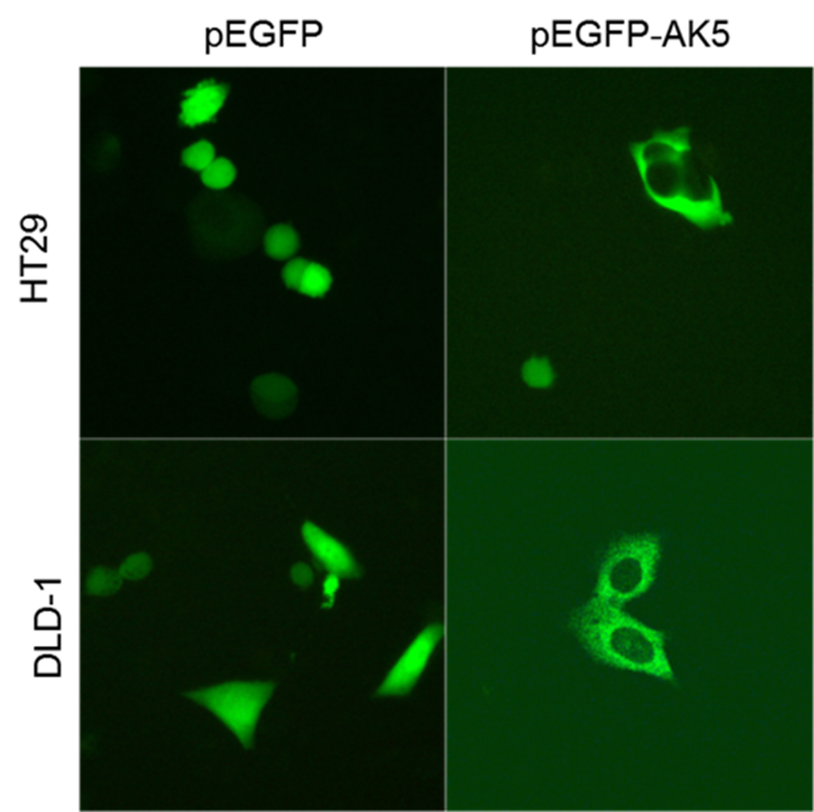
**

**Figure S1. Representation of the AK5 expression pattern in CRC cells.** The expression pattern of AK5-GFP or GFP in cells was visualized using a fluorescence microscope after transfection with pEGFP-AK5 or pEGFP vector in HT29 and DLD-1 cell lines. Original magnification: 400×.


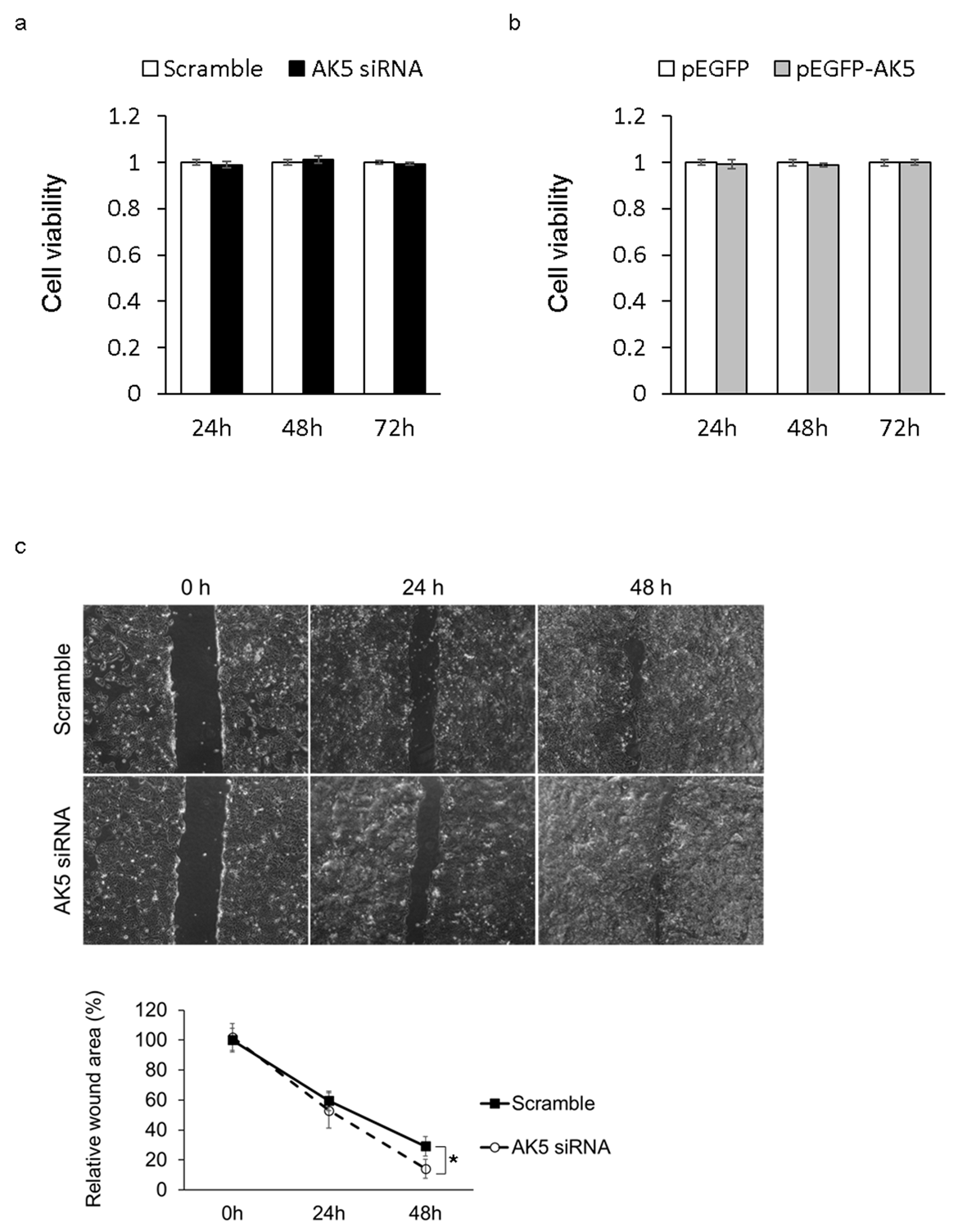


**Figure S2. The effect of AK5 expression on cell viability and migration in DLD-1 cells.** The cell viability of DLD-1 after transfection of AK5 siRNA or pEGFP-AK5 was determined by MTT analysis after 48 hours. (a) The viability of DLD-1 cells showed no difference between AK5 siRNA-transfected cells and scrambled siRNA-transfected cells. (b) The viability of DLD-1 cells showed no difference between pEGFP-AK5 transfected cells and pEGFP transfected cells. (c) The migration of DLD-1 cells was significantly reduced after 48 hours in AK5 overexpression cells transfected with pEGFP-AK5 compared to pEGFP transfected cells. Original magnification: 200×. * P-values of < 0.05 were considered statistically significant.


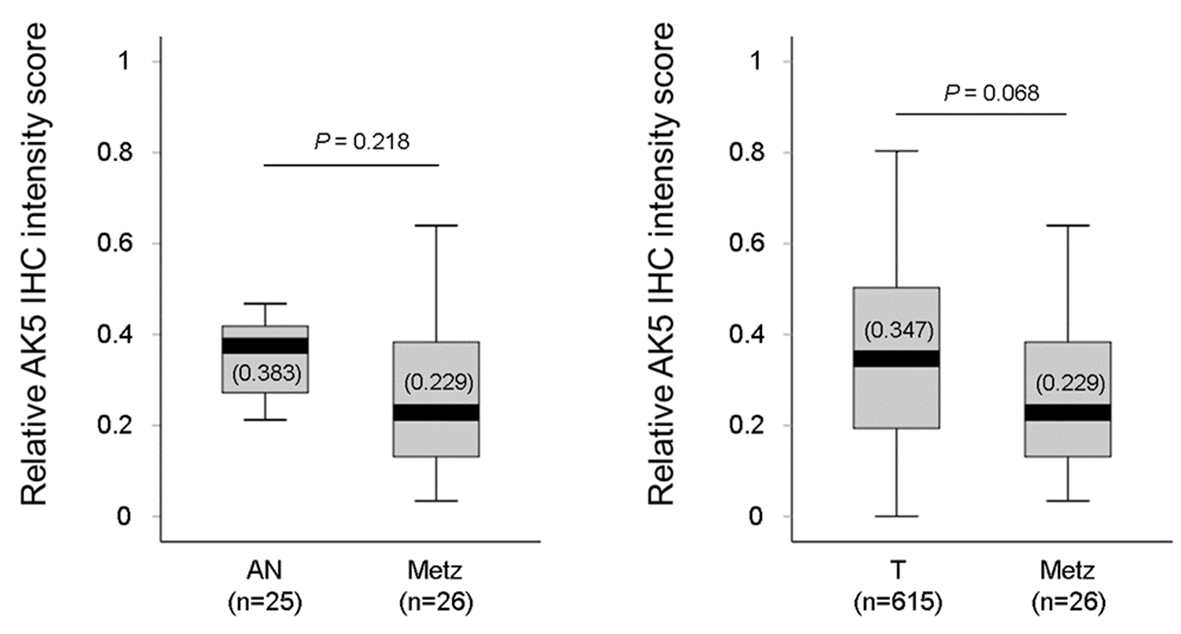


**Figure S3. The results of AK5 IHC in CRC tissues, adjacent normal tissues and metastasis cancer tissues.** Immunohistochemistry results of AK5 in 25 adjacent normal tissues, 615 CRC tissues, and 26 metastasis cancer tissues is quantified using Leica’s Aperio ImagScope program (V12.4.0.5043). The expression of AK5 is relatively decreased in cancer tissues with CRC metastasis. AN, adjacent normal; T, colorectal cancer; Metz; cancer tissue with CRC metastasis.


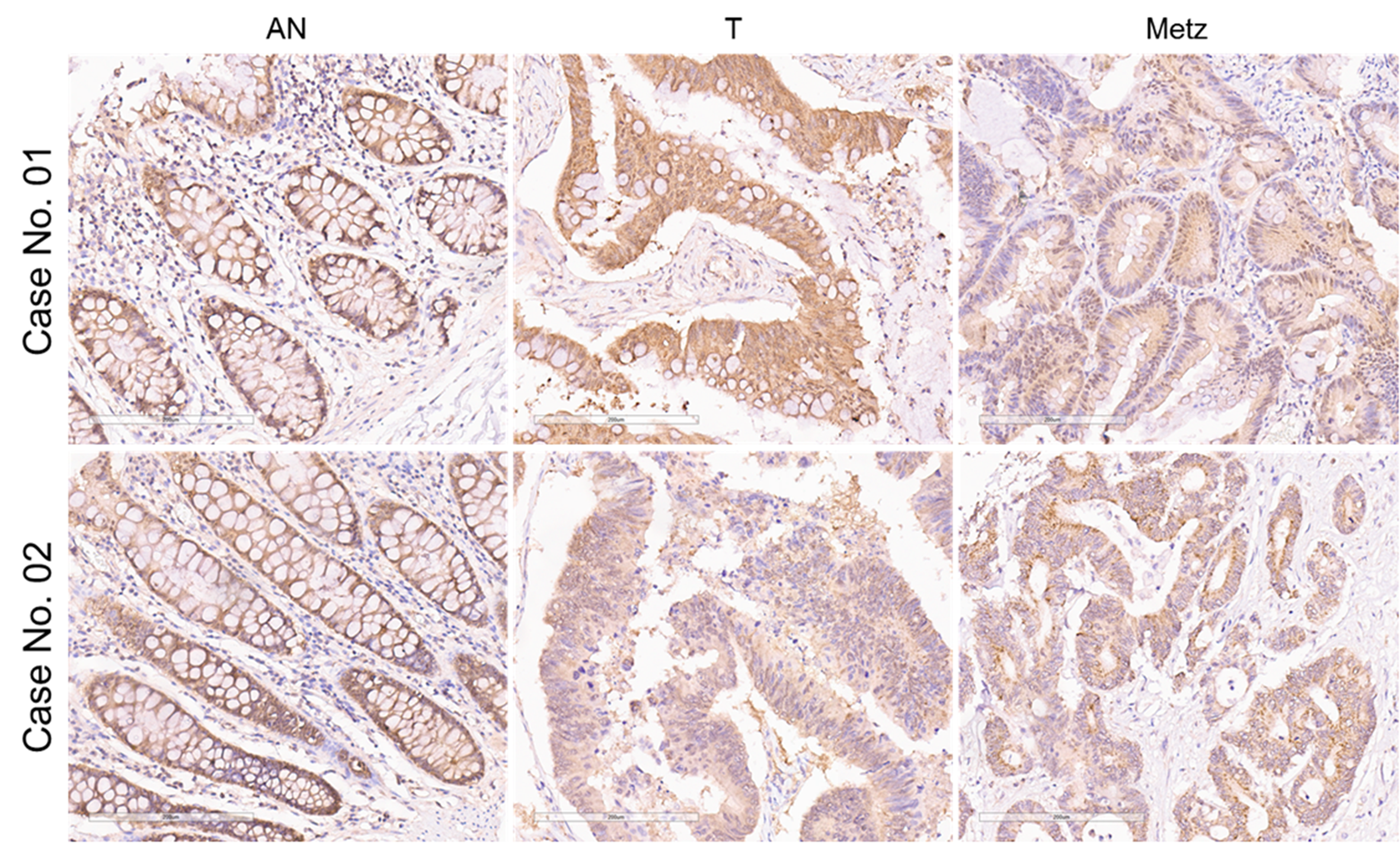


**Figure S4. IHC results of cytoplasmic AK5 expression in CRC, adjacent normal, metastasis cancer tissues.** The expression of AK5 was strongly expressed in adjacent normal tissues, and the AK5 expression tended to gradually decrease in CRC tissues and metastatic tumors. magnification: 100×

a


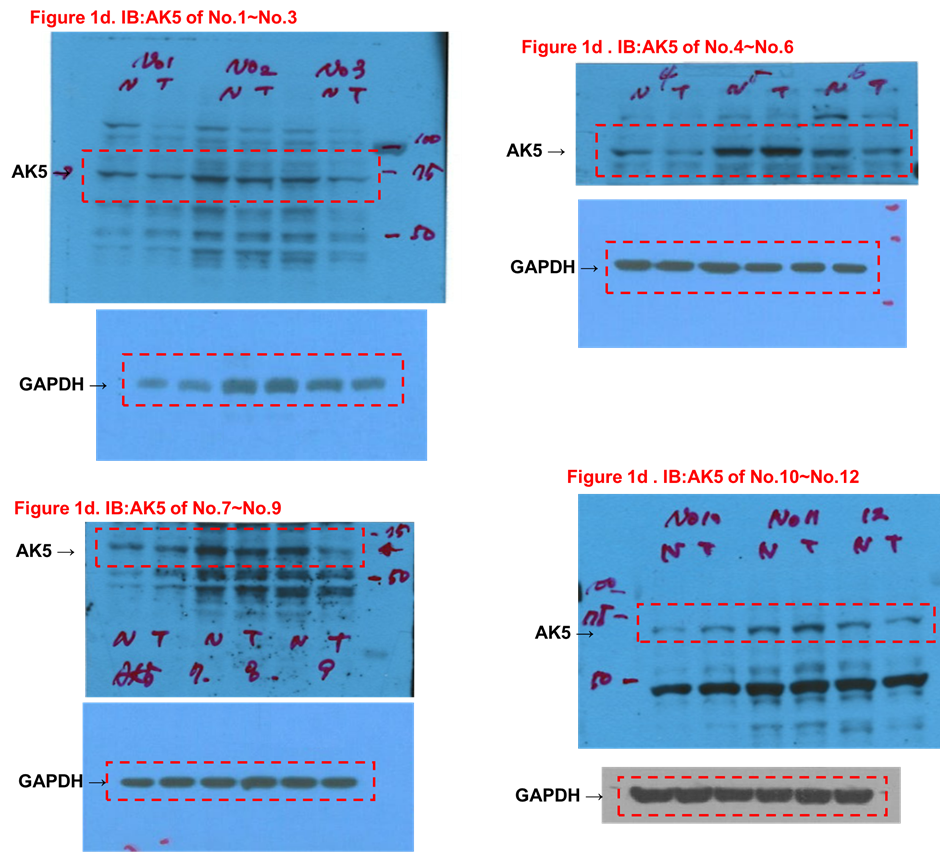


b


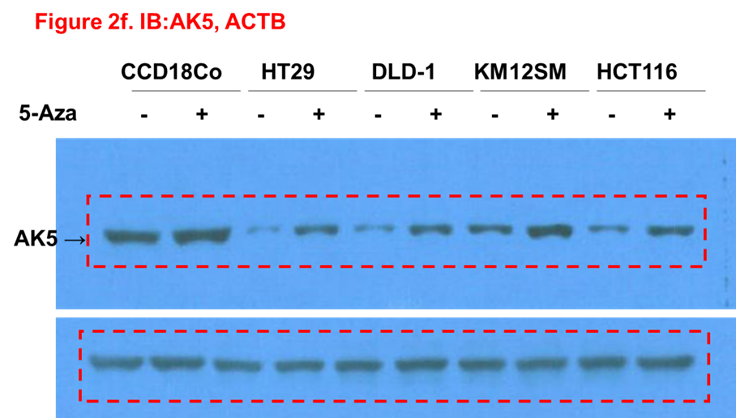


c


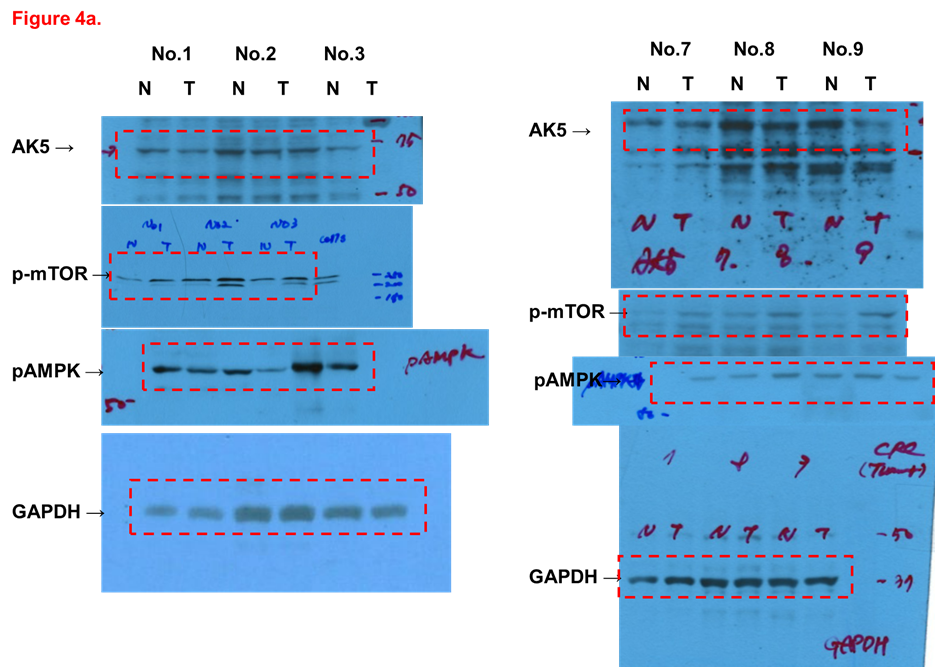


d


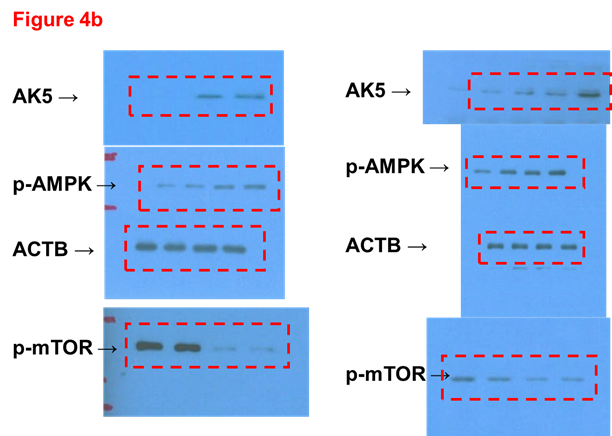


e


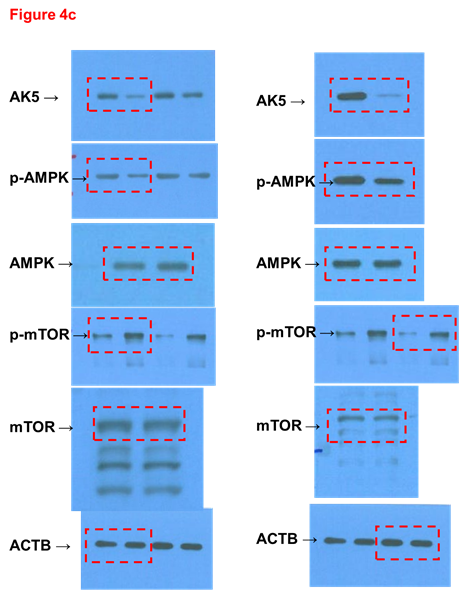


f


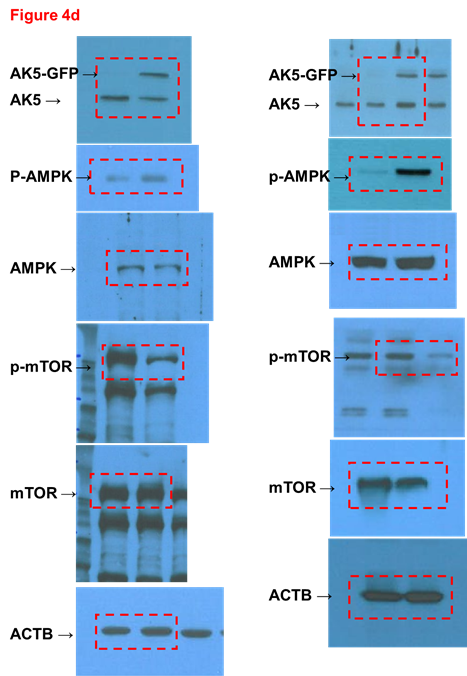


**Figure S5. Full-length or original gel images for Western blot results of target protein.** (a) This is the original gel images of Figure 1d as a results of Western blot analysis of AK5 in CRC tissues and adjacent normal tissues. Anti-AK5 antibody was used Abcam (ab92900) and Epitomics Inc (T3046). Both products were rabbit polyclonal antibodies, and many non-specific bands were detected in human tissues. Therefore, immunoblot analysis was performed by cutting the membrane according to the manufacturer's recommended size. (b) These are the results of protein expression of AK5 in CRC cell lines and normal fibroblast colon cell line. The immunoblot results of AK5 in the cell line showed relatively less non-specific bands than the CRC tissue samples. (c) This is the original gel image in Figure 4a, and is the results of immunoblot analysis for phosphorylated mTOR and AMPK phosphorylation in CRC tissue and adjacent normal tissue. In the case of phosphorylated mTOR, a non-specific band is shown depending on the sample or experimental conditions, whereas in the case of AMPK phosphorylation, a relatively accurate band is shown. (d-f) This is the original gel images in Figure 4b, 4c, and 4d in CRC cell lines after treatment with 5-aza or transfected with AK5 siRNA and AK5 overexpression vectors. They also performed immunoblot analysis by cutting the membrane according to the size recommended by the manufacturer. The red dotted box areas correspond to the cropped portions shown in the main Figure 1, 2, and 4.

**Supplementary Table S1.** Primers for qMSP, real-time PCR, and promoter construct

| **Categories** | **Genes** | **Primer sequences (5'🡪3')** | | **Location** | **Product sizes (bp)** | **Annealing (℃)** | **Gene bank** |
| --- | --- | --- | --- | --- | --- | --- | --- |
| QMSP | AK5 | F ; | GTC GTA TCG AGG GGT ATG ATT AC | (-97~-38) | 59 | 60 | NC_000001.11 |
|  |  | R ; | AAA TAC GAA AAT AAA AAC GAA CAC G |  |  |  |  |
|  | ACTB | F ; | TGG TGA TGG AGG AGG TTT AGT AAG T | (-1645~-1513) | 132 | 60 | NC_000007.13 |
|  |  | R ; | AAC CAA TAA AAC CTA CTC CTC CCT TAA |  |  |  |  |
| Real-time  PCR | AK5 | F ; | TGG TCC AGG AAG TGG AAA GGG | Exon 4-5 | 263 | 60 | NM_174858.3 |
|  |  | R ; | GCC TGG GCA ACA TCT CTT GG |  |  |  |  |
|  | ACTB | F ; | AGA GCT ACG AGC TGC CTG AC | Exon 4-5 | 184 | 60 | NM_001101.5 |
|  |  | R ; | AGC ACT GTG TTG GCG TAC AG |  |  |  |  |
|  | GAPDH | F ; | ACC CAC TCC TCC ACC TTT GA | Exon 8-9 | 101 | 60 | NM_002046.7 |
|  |  | R ; | CTG TTG CTG TAG CCA AAT TCG T |  |  |  |  |
| Promoter  Constructs | AK5 | F ; | CGA GGT ACC CAC GGA CTG CAC ACT CTT CA | (-713~+473) | 1204 | 56 | NC_000001.11 |
|  |  | R ; | CGC AAG CTT GCC CTA CCT CAA AAA GCT GA |  |  |  |  |

**Supplementary Table S2.** Thirty top-ranking genes with CRC-specific hypermethylated CpG site in promoter CpG islands

| **Gene Symbol** | **Reference Seq.** | **Distance to TSS** | **Adj** | **Tumor** | **T-test** | ***p*value** | **FDR *p* value** | **Odds Ratio** | **Fold Change** |
| --- | --- | --- | --- | --- | --- | --- | --- | --- | --- |
| LRRC4 | [NM_022143.3](http://www.ncbi.nlm.nih.gov/entrez/query.fcgi?db=Nucleotide&cmd=search&term=NM_022143.3) | -1167 | 0.045 | 0.461 | -16.077 | 1.072E-16 | 1.639E-13 | 20.518 | 11.035 |
| C1orf165 | [NM_024603.1](http://www.ncbi.nlm.nih.gov/entrez/query.fcgi?db=Nucleotide&cmd=search&term=NM_024603.1) | -397 | 0.044 | 0.417 | -6.965 | 5.534E-07 | 1.549E-05 | 11.445 | 6.104 |
| SLC8A3 | [NM_058240.2](http://www.ncbi.nlm.nih.gov/entrez/query.fcgi?db=Nucleotide&cmd=search&term=NM_058240.2) | -75 | 0.036 | 0.341 | -5.993 | 6.477E-06 | 1.143E-04 | 9.366 | 5.838 |
| SPG20 | [NM_015087.3](http://www.ncbi.nlm.nih.gov/entrez/query.fcgi?db=Nucleotide&cmd=search&term=NM_015087.3) | -209 | 0.069 | 0.445 | -8.168 | 4.691E-09 | 3.101E-07 | 10.276 | 5.796 |
| ZNF272 | [NM_006635.2](http://www.ncbi.nlm.nih.gov/entrez/query.fcgi?db=Nucleotide&cmd=search&term=NM_006635.2) | -46 | 0.039 | 0.256 | -6.207 | 1.828E-06 | 4.140E-05 | 7.167 | 5.442 |
| VAV3 | [NM_006113.3](http://www.ncbi.nlm.nih.gov/entrez/query.fcgi?db=Nucleotide&cmd=search&term=NM_006113.3) | -575 | 0.04 | 0.311 | -7.412 | 1.001E-07 | 3.813E-06 | 7.767 | 5.351 |
| ZNF304 | [NM_020657.1](http://www.ncbi.nlm.nih.gov/entrez/query.fcgi?db=Nucleotide&cmd=search&term=NM_020657.1) | -195 | 0.055 | 0.371 | -6.79 | 1.905E-07 | 6.562E-06 | 8.35 | 5.348 |
| ZNF331 | [NM_018555.4](http://www.ncbi.nlm.nih.gov/entrez/query.fcgi?db=Nucleotide&cmd=search&term=NM_018555.4) | -96 | 0.053 | 0.416 | -6.347 | 2.199E-06 | 4.815E-05 | 9.643 | 5.253 |
| ZNF132 | [NM_003433.2](http://www.ncbi.nlm.nih.gov/entrez/query.fcgi?db=Nucleotide&cmd=search&term=NM_003433.2) | -83 | 0.077 | 0.532 | -6.355 | 9.479E-07 | 2.393E-05 | 12.325 | 5.216 |
| DRD4 | [NM_000797.2](http://www.ncbi.nlm.nih.gov/entrez/query.fcgi?db=Nucleotide&cmd=search&term=NM_000797.2) | -135 | 0.111 | 0.592 | -9.432 | 1.503E-10 | 2.158E-08 | 12.227 | 5.116 |
| ADHFE1 | [NM_144650.1](http://www.ncbi.nlm.nih.gov/entrez/query.fcgi?db=Nucleotide&cmd=search&term=NM_144650.1) | -94 | 0.131 | 0.65 | -15.459 | 1.351E-19 | 7.460E-16 | 12.059 | 4.746 |
| BTG4 | [NM_017589.2](http://www.ncbi.nlm.nih.gov/entrez/query.fcgi?db=Nucleotide&cmd=search&term=NM_017589.2) | -451 | 0.174 | 0.634 | -12.322 | 9.984E-17 | 1.621E-13 | 10.984 | 4.567 |
| CHST10 | [NM_004854.3](http://www.ncbi.nlm.nih.gov/entrez/query.fcgi?db=Nucleotide&cmd=search&term=NM_004854.3) | -188 | 0.077 | 0.527 | -5.963 | 5.954E-06 | 1.069E-04 | 11.745 | 4.462 |
| SDC2 | [NM_002998.3](http://www.ncbi.nlm.nih.gov/entrez/query.fcgi?db=Nucleotide&cmd=search&term=NM_002998.3) | -14 | 0.055 | 0.343 | -7.211 | 3.691E-07 | 1.126E-05 | 6.644 | 4.402 |
| ELOVL4 | [NM_022726.2](http://www.ncbi.nlm.nih.gov/entrez/query.fcgi?db=Nucleotide&cmd=search&term=NM_022726.2) | -193 | 0.088 | 0.418 | -5.668 | 4.462E-06 | 8.448E-05 | 7.429 | 4.322 |
| STK33 | [NM_030906.2](http://www.ncbi.nlm.nih.gov/entrez/query.fcgi?db=Nucleotide&cmd=search&term=NM_030906.2) | -3 | 0.02 | 0.239 | -3.676 | 1.338E-03 | 8.354E-03 | 5.959 | 4.196 |
| SOX5 | [NM_178010.1](http://www.ncbi.nlm.nih.gov/entrez/query.fcgi?db=Nucleotide&cmd=search&term=NM_178010.1) | -484 | 0.051 | 0.325 | -4.855 | 7.874E-05 | 8.763E-04 | 6.373 | 4.089 |
| AKR1B1 | [NM_001628.2](http://www.ncbi.nlm.nih.gov/entrez/query.fcgi?db=Nucleotide&cmd=search&term=NM_001628.2) | -31 | 0.1 | 0.465 | -4.744 | 5.682E-05 | 6.771E-04 | 7.789 | 3.968 |
| KCNQ5 | [NM_019842.2](http://www.ncbi.nlm.nih.gov/entrez/query.fcgi?db=Nucleotide&cmd=search&term=NM_019842.2) | -183 | 0.143 | 0.488 | -9.97 | 1.180E-12 | 4.088E-10 | 6.614 | 3.808 |
| QKI | [NM_006775.1](http://www.ncbi.nlm.nih.gov/entrez/query.fcgi?db=Nucleotide&cmd=search&term=NM_006775.1) | -558 | 0.068 | 0.402 | -4.713 | 1.196E-04 | 1.232E-03 | 7.515 | 3.806 |
| EYA4 | [NM_004100.2](http://www.ncbi.nlm.nih.gov/entrez/query.fcgi?db=Nucleotide&cmd=search&term=NM_004100.2) | -246 | 0.151 | 0.571 | -15.193 | 7.087E-18 | 2.013E-14 | 7.716 | 3.8 |
| NDRG4 | [NM_022910.1](http://www.ncbi.nlm.nih.gov/entrez/query.fcgi?db=Nucleotide&cmd=search&term=NM_022910.1) | -381 | 0.075 | 0.411 | -5.405 | 1.300E-05 | 2.037E-04 | 6.652 | 3.771 |
| TWIST1 | [NM_000474.3](http://www.ncbi.nlm.nih.gov/entrez/query.fcgi?db=Nucleotide&cmd=search&term=NM_000474.3) | -1 | 0.058 | 0.33 | -5.501 | 1.619E-05 | 2.452E-04 | 5.495 | 3.697 |
| COL4A1 | [NM_001845.3](http://www.ncbi.nlm.nih.gov/entrez/query.fcgi?db=Nucleotide&cmd=search&term=NM_001845.3) | -994 | 0.16 | 0.585 | -11.476 | 5.419E-13 | 2.070E-10 | 7.82 | 3.656 |
| FLI1 | [NM_002017.2](http://www.ncbi.nlm.nih.gov/entrez/query.fcgi?db=Nucleotide&cmd=search&term=NM_002017.2) | -275 | 0.072 | 0.307 | -6.75 | 2.220E-07 | 7.421E-06 | 4.993 | 3.652 |
| UNC5C | [NM_003728.2](http://www.ncbi.nlm.nih.gov/entrez/query.fcgi?db=Nucleotide&cmd=search&term=NM_003728.2) | -422 | 0.182 | 0.633 | -13.293 | 4.252E-17 | 7.889E-14 | 8.375 | 3.624 |
| AK5 | [NM_174858.1](http://www.ncbi.nlm.nih.gov/entrez/query.fcgi?db=Nucleotide&cmd=search&term=NM_174858.1) | -355 | 0.071 | 0.323 | -5.399 | 1.752E-05 | 2.610E-04 | 5.443 | 3.623 |
| HCN1 | NM_021072.1 | -372 | 0.126 | 0.474 | -8.694 | 4.161E-09 | 2.844E-07 | 6.341 | 3.565 |
| KHDRBS2 | [NM_152688.1](http://www.ncbi.nlm.nih.gov/entrez/query.fcgi?db=Nucleotide&cmd=search&term=NM_152688.1) | -565 | 0.165 | 0.543 | -10.876 | 2.409E-14 | 1.738E-11 | 6.681 | 3.544 |
| TFPI2 | [NM_006528.2](http://www.ncbi.nlm.nih.gov/entrez/query.fcgi?db=Nucleotide&cmd=search&term=NM_006528.2) | -118 | 0.169 | 0.52 | -9.063 | 7.763E-12 | 1.902E-09 | 6.374 | 3.535 |

**Supplementary Table S3.** Clinicopathological characteristics of CRC patients and methylation status and mRNA expression levels of AK5

| Characteristics | No. of cases | Methylation status of *AK5*  (PMR, %) | | mRNA expression levels of *AK5* (%) | |
| --- | --- | --- | --- | --- | --- |
|  |  | Median (range) | *p*-Value | Median (range) | *p*-Value |
|  |  |  |  |  |  |
| Normal colon | 11 | 20.83 (± 2.65) | < 0.001^†^ | 28.18 (± 4.51) | 0.06 |
| Adjacent normal | 105 | 42.26 (± 1.72) |  | 8.55 (± 2.43) |  |
| Colorectal cancer | 105 | 56.72 (± 4.33) |  | 2.14 (± 0.53) |  |
| Age (years) |  |  | 0.447 |  | 0.683 |
| ≤ 60 | 28 | 63.30 (± 7.23) |  | 3.03 (± 1.13) |  |
| > 60 | 77 | 51.17 (± 5.30) |  | 1.87 (± 0.59) |  |
| Gender |  |  | 0.793 |  | 0.409 |
| Female | 39 | 50.31 (± 8.53) |  | 2.29 (± 0.73) |  |
| Male | 66 | 59.23 (± 4.75) |  | 1.72 (±0.71) |  |
| Differentiation |  |  | 0.893 |  | 0.893 |
| Well | 29 | 60.39 (± 10.39) |  | 2.26 (± 1.10) |  |
| Moderate | 73 | 50.98 (± 4.56) |  | 1.86 (± 0.63) |  |
| Poorly | 3 | 93.80 (± 22.91) |  | 7.71 (± 0.18) |  |
| Location |  |  | 0.454 |  | 0.749 |
| Colon | 72 | 69.88 (± 5.32) |  | 9.82 (± 0.60) |  |
| Rectum | 33 | 62.86 (± 7.49) |  | 11.29 (± 1.06) |  |
| TNM Stage |  |  | 0.065 |  | 0.1 |
| Ⅰ,Ⅱ | 46 | 61.03 (± 8.16) |  | 2.02 (± 1.03) |  |
| Ⅲ,Ⅳ | 59 | 51.84 (± 4.20) |  | 2.14 (± 0.47) |  |
| Metastasis |  |  | 0.809 |  | 0.568 |
| No | 92 | 68.07 (± 4.78) |  | 2.14 (± 0.59) |  |
| Yes | 13 | 64.87 (± 9.21) |  | 1.75 (± 0.67) |  |
| Size (cm) |  |  | 0.805 |  | 0.892 |
| ≤ 6 | 79 | 53.79 (± 5.35) |  | 2.14 (± 0.60) |  |
| > 6 | 26 | 60.63 (± 6.59) |  | 2.68 (± 1.11) |  |
|  |  |  |  |  |  |

Statistical significance was evaluated by analysis of variance (ANOVA). ^†^*p-*values < 0.05 were considered statistically significant. PMR: Percentage of methylated reference; TNM: tumor, lymph nodes and metastasis.
